# Effectiveness of a Malaysian Media Intervention Workshop: Safe Reporting on Suicide

**DOI:** 10.3389/fpsyg.2021.666027

**Published:** 2021-12-15

**Authors:** Jane Tze Yn Lim, Qijin Cheng, Yin Ping Ng, Kai Shuen Pheh, Ravivarma Rao Panirselvam, Kok Wai Tay, Joanne Bee Yin Lim, Wen Li Chan, Amer Siddiq Amer Nordin, Hazli Zakaria, Sara Bartlett, Jaelea Skehan, Ying-Yeh Chen, Paul Siu Fai Yip, Shamsul Azhar Shah, Lai Fong Chan

**Affiliations:** ^1^Department of Psychiatry, Faculty of Medicine, National University of Malaysia (UKM), Kuala Lumpur, Malaysia; ^2^Department of Social Work, The Chinese University of Hong Kong, Sha Tin, Hong Kong SAR, China; ^3^Psychiatry Specialty, Pantai Hospital Penang, Bayan Lepas, Malaysia; ^4^Department of Psychology and Counselling, Faculty of Arts and Social Sciences, University Tunku Abdul Rahman, Kampar, Malaysia; ^5^Department of Psychiatry and Mental Health, Hospital Miri, Miri, Malaysia; ^6^School of Media, Languages & Cultures, Faculty of Arts & Social Sciences, University of Nottingham Malaysia, Semenyih, Malaysia; ^7^Nottingham University Business School, University of Nottingham Malaysia, Semenyih, Malaysia; ^8^Department of Psychological Medicine, Faculty of Medicine, Universiti Malaya, Kuala Lumpur, Malaysia; ^9^Malaysian Psychiatric Association, Kuala Lumpur, Malaysia; ^10^Everymind, Newcastle, NSW, Australia; ^11^Taipei City Psychiatric Center, Taipei City Hospital, Taipei City, Taiwan; ^12^Institute of Public Health and Department of Public Health, National Yang-Ming University, Taipei City, Taiwan; ^13^Department of Social Work and Social Administration, The University of Hong Kong, Hong Kong, Hong Kong SAR, China; ^14^Department of Community Health, Faculty of Medicine, National University of Malaysia (UKM), Kuala Lumpur, Malaysia

**Keywords:** Malaysian, media, workshop, safe reporting, suicide

## Abstract

**Background:** Suicide remains an important cause of premature deaths and draws much media attention. However, unsafe reporting and portrayal of suicides by the media have been associated with increased risk of suicidal behavior. Current evidence suggests that media capacity-building could potentially prevent suicide. However, there are still knowledge gaps in terms of a lack of data on effective strategies for improving awareness and safe reporting of suicide-related media content. This study aims to investigate the effectiveness of a workshop conducted with members of the media community on the safe reporting of suicide-related content.

**Methods:** An interventional single-arm pre and post pilot study was conducted on a sample of the Malaysian media community recruited through purposive and snowball sampling. The media safe reporting workshop was conducted by a suicide prevention expert with a media industry background. Thirty participants completed a self-reported evaluation questionnaire on their awareness and knowledge of reporting on suicide-related media content; before and after the interventional workshop.

**Results:** There was a significant difference between the total scores before and after the intervention, with a large effect size. Post-intervention scores were significantly improved in 8 items, namely those related to the reporting of: (i) the content of any suicide note; (ii) headlines with methods of suicide; (iii) headlines with the location of suicide; (iv) cases of suspected suicide despite the unconfirmed cause of death; (v) suicide news to cater to readers’ interests; (vi) cause of suicide; (vii) details of the location of suicide; and (viii) the negative impact to media community when reporting suicide stories. In particular, there was an improvement in the majority of items for people from the media community with no lived experience of suicidal behavior.

**Conclusion:** The media safe reporting workshop is a potentially effective intervention for improving awareness and knowledge measures relating to safe reporting on suicide among the media community, with a more pronounced effect in those without lived experience of suicidal behavior. Limitations in the sample size, generalizability, short-term evaluation, and lack of a control group warrant future larger, longer-term controlled, and more representative studies.

## Introduction

Suicide is a public health concern that draws media attention worldwide. Globally every year, approximately 800,000 people die by suicide (WHO, 2016). The impact of suicide is profound. For every individual who dies by suicide, an estimated average of 135 people are markedly affected (Cerel et al., 2019). The suicide-bereaved have been found to experience significant negative trauma reactions, stigmatization, mental disorders, and suicidal behavior (Cerel et al., 2005). Suicide has been known to be the result of a complex dynamic interplay between numerous contributing factors, with evolving knowledge of potential risk factors. For one, childhood maltreatment has been significantly associated with non-suicidal self-injury and suicide attempts (Serafini et al., 2017a). Other factors such as extreme sensory processing patterns have significant correlations with affective disorders, with the prevalence of lower registration in those with higher hopelessness (Serafini et al., 2017b), often leading to suicidal behavior. Past studies have considered multi-level and multi-faceted approaches to combat suicide including universal interventions aimed at different populations; as well as more selective interventions tailored for high risk groups and those with suicidal behavior (Institute of Medicine Committee on Prevention of Mental Disorders, 1994; Department of Health Tag, 2007; WHO, 2016).

The impact of the reporting and portrayal of suicide in the media has become an increasing public health concern (Sisask and Värnik, 2012). Media suicide stories or reports, especially those involving celebrities; which contain explicit descriptions of the method and location of the suicide, repeated sensationalized coverage, as well as the portrayal of suicide as a solution to life problems, have been observed to be associated with increased risk of suicidal behavior (Pirkis et al., 2006, 2018; Niederkrotenthaler et al., 2009, 2012, 2020). The rate of suicide increased by 9.85% during the months following the death by suicide of the famous Hollywood comedian and actor Robin Williams (Fink et al., 2018). The increase in suicide attempts is proportionate to the publicity generated by wide (unsafe) media coverage of suicide incidents (Hassan, 1995). Inappropriate reporting and sensationalized news glorifying the suicidal act of a person or celebrity could lead to suicide contagion and ‘copycat suicides’ among individuals with pre-existing vulnerabilities of suicidal behavior (Phillips, 1985; Hassan, 1995). While inappropriate reporting can pose harm to the concerned community, responsible media reporting has the potential to reduce the risk of suicide (Phillips, 1985; Hassan, 1995; Samaritans, n.d.). For example, the less sensationalized reporting of the suicide of U.S musician Kurt Cobain, emphasizing its impact on mental health as well as the availability of support services for those similarly afflicted sent positive messages to concerned communities (Jobes et al., 1996). Simple messages on the preventability of suicide and availability of help services in media campaigns led to increased calls to helplines (Oliver et al., 2008; Jenner et al., 2010). Media reports that highlighted adaptive coping or the personal mastery of problems when facing a crisis were inversely associated with suicide rates (Niederkrotenthaler et al., 2010).

Amid this growing body of evidence supporting the potential positive influence of the media in preventing suicide, guidelines for media reporting on suicide have changed the way suicide-related news is reported, meaning it is sensitive to the public and, in turn, associated with a reduction in suicide rates (Bohanna and Wang, 2012). However, the effectiveness of such guidelines requires consultation and collaboration with the media community (Tully and Elsaka, 2004; MediaWise, 2006; Bohanna and Wang, 2012; Duncan and Luce, 2020b). Intersectoral collaboration amongst all stakeholders in suicide prevention is thus crucial, and good media collaboration and training have been proposed as strategies for suicide prevention campaigns. While efforts have been made to integrate the education on appropriate reporting of suicide into journalism curricula, such as the Response Ability Project, a collaboration between mental health professionals and journalism educators at Australian universities (Skehan et al., 2009), there is also a need for continued monitoring and training to ‘refresh’ journalists’ knowledge in order for adherence to guidelines to be sustained (Niederkrotenthaler and Sonneck, 2007; Bohanna and Wang, 2012; Cheng et al., 2014). In Malaysia, changes in the media landscape have provided alternative ways for people to assess information; through new media journalism, the internet, social media, and blogs (Weiss, 2013; Mustaffa et al., 2017; Taibi and Na, 2020). While media reporting guidelines were published in 2004 (Ministry of Health Malaysia, 2004), reporting practices have seen little improvement. There is a poor level of awareness and lack of responsible reporting in the Malaysian media (Johari et al., 2017; Victor et al., 2019; Fong, 2021; Ng et al., 2021a). The prevalence of suicide in Malaysia was 6–8 per 100,000 population per year (Armitage et al., 2015). Hence, there is a clear need for collaborative engagements with stakeholders of safe media reporting including the media community (Johari et al., 2017; Chan et al., 2018).

In the European context, a workshop intervention with Swiss journalists was shown to increase participants’ self-efficacy and attitudes about safe reporting (Scherr et al., 2019). In Hong Kong, continuous engagement with media professionals through knowledge exchange and monitoring efforts saw evidence of reduced intensity and sensationalism in suicide news reports (Law et al., 2019).

Our study also contributes to the body of knowledge in this area by taking an objective approach in evaluating the effectiveness of a workshop for the media community on safe media reporting. In particular, this study assessed the level of awareness and knowledge of key aspects of safe suicide reporting practices, before and after the workshop intervention, amongst a sample of Malaysian media community professionals.

## Materials and Methods

An interventional single-arm pre and post-pilot study were conducted among a sample of the Malaysian media community and professionals, recruited through purposive and snowball sampling. We hypothesized that there would be significant differences in terms of the self-reported scores of awareness and knowledge of safe suicide reporting among the media community and professionals before and after participation in the media intervention workshop. Our use of the term media community refers to media professionals, media academics and media journalism students who may be print/broadcast/digital media director, content producer, journalists, editors, sub-editors, news stringers, newscasters, columnists, bloggers, video-bloggers (or vloggers) working on one or more media platforms including newspapers, magazines, television, radio and online/social media. News platforms in Malaysia can be found in different languages – Malay, English, Chinese, and Tamil. The inclusion criteria were: (i) aged 18 years old or older, (ii) a member of the media community and professionals, (iii) sufficiently proficient in the English language, (iv) not clinically depressed based on a Patient Health Questionnaire-9 (PHQ-9) score of less than 10, (v) had not had active suicidal thoughts or plans in the 2 weeks prior to the workshop, any suicide attempt in the 6 months prior to the workshop or been bereaved by suicide in the 6 months prior to the workshop. The exclusion criteria were current suicidal ideation within the past 2 weeks i.e., positive screen for suicidal ideation, or significant depressive symptoms with total scores in a Patient Health Questionnaire (PHQ-9) of 10 and above (Kroenke et al., 2001; Sherina et al., 2012) or persons who had attempted suicide or were bereaved by suicide in the past 6 months.

**FIGURE 1:**
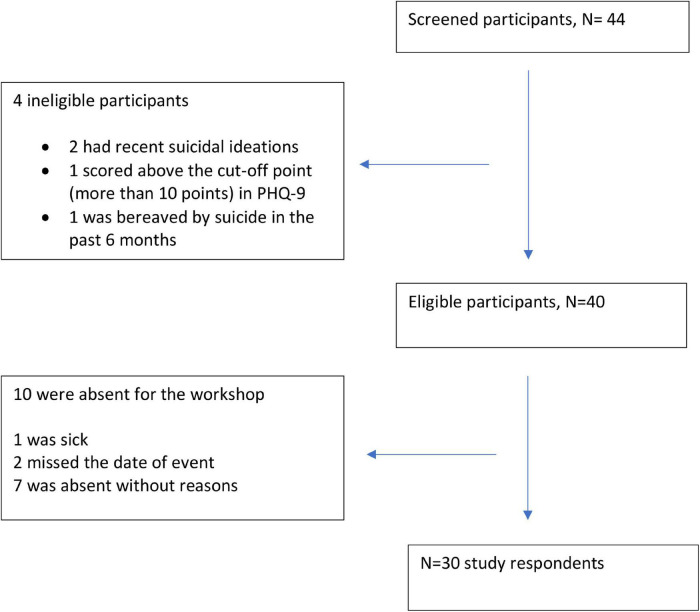
Summary of study respondents.

The required sample size for this study is a minimum of 30 participants, estimated based on the rule of thumb for a pilot study (Hooper, 2019) and G*Power software (Faul et al., 2007) for a power of 80% with α = 0.05, two-tailed and an estimated effect size of 0.5. A total of 40 participants were eligible for inclusion in the study (Figure 1). On-site psychological support was provided by a volunteer mental health professional who was not a member of the research team. Any participant with a positive screen in the post-evaluation modified Patient Health Questionnaire (PHQ) who required psychological support after the media workshop would be attended to by the mental health professional with further mental health services offered if needed.

The intervention, which is the media safe reporting workshop, was conducted on February 20, 2019, by one of the research team members, QC who is a suicide prevention expert with a media background. The 2-h workshop consisted of core topics on media guidelines, fundamental principles in suicide reporting as well as specific recommendations and cases for discussion (Table 1), adapted from a book on recommendations on suicide reporting (Yip et al., 2015). Participants were required to answer individually, 16 questions in the evaluation questionnaire that assess participants’ awareness and knowledge of key aspects of safe suicide reporting (Table 2). The evaluation questionnaire was adapted from an updated version of a questionnaire used in the Response Ability Project (Skehan et al., 2009). The pre and post-intervention responses of each participant for the same evaluation questionnaire were matched using an anonymous code. Each question was scored with a Likert scale of 0–5; with a total sum of 80. Higher scores correspond to higher levels of awareness and knowledge of safe suicide reporting.

**TABLE 1:** The media safe reporting workshop.

Total hours	Core topics
Part I: 1 hour	Part 1: Introducing the overarching media guidelines 1) A brief history of media guidelines on suicide news reporting 2) The need to update media guideline 3) Fundamental principles in responsible suicide reporting a) Protect privacy and consider the risks borne by vulnerable individuals. b) Take the opportunity to educate the public about mental health and suicide prevention c) Practice self-care in the community of media professionals
Part II: 1 hour	Part 2 A) Specific recommendations – Selective reporting and editorial considerations B) Case discussion

**TABLE 2:** Participants’ pre post intervention effects.

No.	Question items	Pre-intervention scores		Post-intervention scores		Intervention effect		
				
		Mean	SD	Mean	SD	T	*df*	*p* value
1	Reports about suicide should not mention specific details about the method of suicide.	4.07	0.944	4.33	0.884	1.246	29	0.223
2	Reports about suicide should include graphic details of suicide.	4.53	0.629	4.57	0.568	0.372	29	0.712
3	Reports about suicide should include contents of any suicide note.	3.67	1.093	4.23	0.858	3.084	29	0.004*
4	A celebrity suicide should be reported in detail.	4.13	0.776	4.07	0.785	−0.441	29	0.662
5	The word “suicide” should not be included in the headline or search term in reporting cases of suicide.	2.93	0.907	3.20	1.095	1.490	29	0.147
6	Headlines should include the method of suicide.	4.07	0.884	4.48	0.688	2.703	28	0.012*
7	Headlines should include the location of suicide.	3.53	1.106	4.07	0.828	3.764	29	0.001*
8	A suspected suicide should be reported as suicide even before the cause of death is confirmed.	4.20	0.961	4.63	0.615	3.496	29	0.002*
9	Media portrayal of suicide can lead to copy-cat suicide.	4.27	0.640	4.40	0.855	0.941	29	0.354
10	Suicide-related stories are written to cater to readers’ interest.	2.66	1.111	3.21	1.207	2.339	28	0.027*
11	Stories of recovery from suicidal crisis can be helpful.	4.40	0.132	4.43	0.124	0.372	29	0.712
12	Media should portray suicide as an acceptable response to life’s problems.	4.33	0.205	4.37	0.212	0.183	29	0.856
13	A cause for suicide should always be reported in cases of suicide.	2.70	1.119	3.23	1.073	3.247	29	0.003*
14	Including help-seeking information to stories about suicide is not helpful.	4.27	0.980	4.50	0.682	1.882	29	0.070
15	Reports should include the location of a suicide death.	3.00	0.947	3.70	1.055	3.881	29	0.001*
16	People in the media can be negatively affected when reporting on suicide.	3.97	0.850	4.23	0.817	2.112	29	0.043*
	Total scores (*d* = 0.9)	60.50	8.249	65.60	8.122	4.936	29	<0.001*

*p < 0.05 significant findings.

*d is Cohen’s d.

The data were then analyzed using the Statistical Package for the Social Sciences (SPSS) software, version 26.0. Participants’ baseline characteristics were analyzed using Kruskal-Wallis and Mann Whitney tests. The pre- and post-intervention scores for the evaluation questionnaires were analyzed using a paired *t*-test. Cohen’s *d* was used as the measure of effect size. Cohen’s *d* value of 0.20, 0.50, and 0.80 indicate small, moderate, and large effect sizes respectively (Cohen, 2013). For each item in the evaluation questionnaire that yielded significant findings (*p* < 0.05), further analysis using the Wilcoxon Signed Ranks Test was used for examining the group characteristics of the participants. These related to types of media portals, their roles and job scope, length of experience, and frequency of encounter with suicide news, awareness of the existence of media guidelines and their adherences, and whether they have significant others with suicidal behavior (Supplementary Material).

## Results

Thirty participants (75% of invited) completed the questionnaires before and after the intervention. Four participants were excluded; 1 scored more than 10 points in the PHQ-9, another was bereaved by suicide in the past 6 months, and 2 had recent suicidal ideations (Figure 1). Nearly three quarters 73.3% (*n* = 22) are media practitioners with 23.3% (*n* = 7) comprising media academics and students. More than 80% (83.3%, *n* = 25) were in full-time work or study and 40% (*n* = 12) had professional experience of more than 10 years. All the participants had no prior exposure to any form of training on safe suicide reporting. Of the types of media portals, 46.7% (*n* = 14) were strictly online portals. Sixty-three point three percent (*n* = 19) of them were aware of the existence of any forms of media guidelines for safe suicide reporting, 30% (*n* = 9) knew of the existence of Malaysian media guidelines. About one-third of the participants (36.7%, *n* = 11) had significant others with suicidal thoughts in the past, while 10% (*n* = 3) had significant others who made a suicide attempt in their lifetime. The pre-intervention scores for awareness and knowledge of safe reporting were not significantly different concerning all characteristics, except one. A Mann-Whitney test indicated that participants who were not aware of the existence of a Malaysian media guideline on suicide reporting has higher median scores of pre-intervention awareness and knowledge of safe reporting (Mdn = 64) than those who were aware of its existence (Mdn = 56), *U* = 50.50, *p* = 0.046.

On the effect of intervention, there was a statistically significant difference between the total scores of awareness and knowledge of safe reporting between pre- and post-intervention with large effect size [*t* (29) = 4.936, *p* < 0.001, *d* = 0.9]. There are 8 items for which scores were significantly improved post-intervention (Table 2); and were related to knowledge on (i) reports of contents of any suicide note [*t* (29) = 3.804, *p* < 0.01], (ii) headlines with methods of suicide [*t* (28) = 2.703, *p* < 0.05], (iii) headlines with location of suicide [*t* (29) = 3.764, *p* < 0.01], (iv) cases of suspected suicide despite unconfirmed cause of death [*t* (29) = 3.496, *p* < 0.01], (v) suicide news to cater to readers’ interests [*t* (28) = 2.339, *p* < 0.05], (vi) reports on cause of suicide [*t* (29) = 3.247, *p* < 0.01], (vii) reports on location of suicide [*t* (29) = 3.881, *p* < 0.01] as well as (viii) negative impact to media community when reporting on suicide [*t* (29) = 2.112, *p* < 0.05].

For all these significant items (see Supplementary Material), the common group characteristic factor in the improvement of post-intervention scores was the media community with no lived experience. Media communities with no lived experience scored significant improvement scores (*p* < 0.05) in all 8 items as compared to those with lived experience. Those in the media community who worked full-time as compared to part-time scored significantly different (*p* < 0.05) in 7 out of 8 significant items, the 1 exclusion item was “suicide news to cater to readers’ interests.” Other group characteristic factors were online news portals and media community who were aware of the existence of any forms of media guidelines for safe suicide reporting; both with significant findings (*p* < 0.05) in 6 out of 8 items. No participants screened positive in the modified post-evaluation Patient Health Questionnaire.

## Discussion

Safe suicide reporting was identified as one of the main strategies for suicide prevention (Niederkrotenthaler et al., 2010, 2012, 2020; Bohanna and Wang, 2012; Sisask and Värnik, 2012). Our study highlighted the positive role of a workshop intervention in training the media community about safe suicide reporting. The improvement was evidenced in the awareness and knowledge domains of safe reporting of suicide after the 2-h media workshop, in particular pertaining to knowledge of inappropriate reporting of headlines with methods and location of suicide, the inclusion of any suicide note, cause and details of suicide location in the content of reports, as well as negative impact of reporting unconfirmed cases and to cater for readers’ interest. There was also an increase in awareness of the negative impact on the media community when reporting suicide stories. These overall findings are also supported by another study (Scherr et al., 2019) on positive outcomes of workshop intervention among Swiss media professionals in relation to their self-efficacy and attitudes in suicide reporting. One limitation to safe practice may be contributed by the lack of knowledge and expertise or skills surrounding the process of reporting. Our study provides theoretical knowledge on specific components of safe suicide reporting such as harmful descriptions of suicide and protective suicide-prevention (Ng et al., 2021b) in line with Papageno and Werther’s discussion of effect in media suicide reporting (Phillips, 1985; Niederkrotenthaler et al., 2010). Knowledge of these components was evidenced to show significant improvement after our media workshop.

Other useful components may include the introduction of the responsible suicide reporting (RSR) model (Duncan and Luce, 2020b), which utilizes three key aspects, namely: (i) typology of suicide story, (ii) four ethical rules in reporting; and (iii) Standard of Moderation. The authors described a five narrative model (event-driven, post-judicial, tribute-drive, anniversary, and action-as-memorial) for suicide news and their implications in each story type. The narrative model provides a means for journalists to reflect on their ethical responsibility and minimizes unsafe reporting through four ethical rules: not to sensationalize, stigmatize, glorify, and gratuitously (mnemonic: SSGG) report suicide-related stories in the media. Lastly, the RSR model emphasizes the standard of moderation (harm minimization to bereaved, safe information, tone and language used, the responsible gathering of content, and providing information on the support available), which encourages journalists to critically reflect on in their reporting process. A workshop teaching media students using the RSR model through storytelling and problem-based learning reported greater understanding and familiarity with the practical actions for responsible reporting (Duncan and Luce, 2020a).

Some of the items that improved significantly post-intervention came from participants who were not aware of the existence of Malaysian media guidelines. Various studies worldwide have shown that even with the existence of media guidelines, many challenges arise in implementing them (Gandy and Terrion, 2015; Yaqub et al., 2020). Although guidelines are viewed as useful information, the reality of media culture and its core values in maintaining press freedom and the rights to discuss issues deemed as public interest seemed to be in contrast to the ‘restrictive’ style of suicide reporting. The perception that guidelines are developed via one-way communication with questionable relevance in the mainstream media, since the increase in blogs and social media made implementing guidelines all the more challenging (Gandy and Terrion, 2015). The coverage of suicide stories in episodic, event-centered frames is not motivated for educational purposes and journalists may take an approach of these as being social issues, and not comply with media guideline recommendations. While some struggle with professional values and social responsibilities, others may hold deeply to their journalistic values to ‘tell it all’ (Yaqub et al., 2020). These factors, in addition to low penetrance and dissemination of information and knowledge, are similarly postulated to be the reasons why our local media guidelines are left unheeded. There are also differing perspectives at the individual level on what it is important to report (Pirkis and Machlin, 2013). As such, awareness of the existence of any media guidelines does not automatically translate to compliance with its content.

Most participants with or without lived experience agreed that reporting had a negative impact on them, with a mean score of 3 and above in the pre-evaluation questionnaire. Further to this, media workers with lived experiences of suicide may provide insights into its effects in the workshop, providing assessable information via their real-life experience. They would most likely be aware of the exact location of suicide and the reasons (cause) behind the death of their loved ones and thus be able to perceive that it was inappropriate (unsafe) to report this information. These items on the cause and location of suicide had statistical improvement in the post-intervention mean score on it being unsafe to report. However, the overall effectiveness of the media workshop is more pronounced in those without lived experience of suicidal behavior. The impact of the media workshop on the participants with lower exposure to suicidal behavior potentially addressed the limitations in their understanding of the impact of suicide contagion and the importance of safe reporting. Hence, the direction of future media workshops could be targeted to benefit more people with no lived experience.

To the best of our knowledge, this is the first study to highlight that the knowledge and intrinsic factors covered by a media workshop may improve the effectiveness of training the media community and professionals on the safe reporting of suicide-related content. The other strength of our study is the benefit of better participant engagement when the media workshop is led by someone (QC) with dual expertise in suicide prevention and industry experience. It is safe to conduct the media workshop with participants pre-screened by the post-intervention modified Patient Health Questionnaire (PHQ), indicating that discussing suicide in the workshop was unlikely to cause psychological distress to the participants. However, whilst all 15 items in our evaluation questionnaire showed improvement in mean score post-intervention, there was one item that had a notably lower mean score post-intervention, though it was not statistically significant. This item is related to detailed reporting of a celebrity suicide, which may have not been highlighted adequately in the workshop, possibly due to its infrequent occurrence in our local setting. Nevertheless, future workshops should work toward addressing this pertinent point.

There were a further eight items for which participants’ scores on their awareness and knowledge of safe reporting of suicide improved after participating in the media intervention workshop. They were related to reporting on: (i) the specific details of the method of suicide; (ii) the inclusion of graphic details of the suicide; (iii) detailed reports of celebrity suicide; (iv) use of the word “suicide” in headlines or search terms; (v) media portrayal of suicide leading to copy-cat suicide; (vi) stories of recovery; (vii) portrayal of suicide as an acceptable response to life’s problems; and (viii) the inclusion of help-seeking information. However, the difference between pre and post-scores were not statistically significant, possibly due to the type II error contributed by the study’s small sample size.

## Limitations

One of the main limitations of our study is the small sample size, which predominantly included English-language media in Malaysia. The participants were not randomized, and no control group was used. Future research could use randomized trials for better comparison and to rule out other factors that may be involved in such a workshop. Our study focused on awareness and knowledge of safe reporting on suicide-related content in the media. Suicide is a complex and multifactorial phenomenon, and the scope of our study does not address other equally important challenges facing the media or the news reporting process, such as competing professional values and perceived social responsibilities to report the truth with full disclosure of information (Yaqub et al., 2020). Furthermore, due to time factors and lack of resources, the findings in our study were limited to short-term evaluation. Further studies should examine whether this knowledge is better able to be translated into safe journalistic practice when reporting on suicide.

The evaluation questionnaire in this study, which was adapted and modified from The Response Ability Project (Skehan et al., 2009), was not locally validated. The Response Ability Project (MediaWise, 2006) is a collaboration between mental health professionals and journalism educators in Australia which seeks to exert a positive influence on the education of journalists, enabling them to appropriately respond to and report on issues relating to suicide and mental illness by incorporating these core skills into the journalism curricula. The Mindframe National Media Initiative in Australia, under which the Response Ability project sits, has expanded to support and develop resources for journalists on reporting suicide and mental illness. Before the study, a face validity of the questionnaire was performed in consultation with experts from Mindframe (Everymind, 2021), which utilized the original questionnaire.

## Conclusion

Our study showed preliminary evidence that a media intervention workshop is a potentially effective way of improving awareness and knowledge of safe suicide reporting among media professionals. The workshop has a more pronounced effect on those who do not have lived experiences of suicidal behavior. The implications of our study findings include enhancing advocacy efforts and capacity building of media community professionals as a means to improve the safety of suicide reporting as a suicide prevention strategy. Future larger, randomized controlled, and more representative studies would be welcome to investigate the effectiveness of such workshops in the longer term, as well as studies examining how such workshops influence the ways in which suicide-related content is reported and exploring the potential impact on suicide rates.

## Data Availability Statement

The original contributions presented in the study are included in the article/Supplementary Material, further inquiries can be directed to the corresponding author.

## Ethics Statement

The studies involving human participants were reviewed and approved by University Kebangsaan Malaysia Medical Centre Ethics Committee (UKMPPI/111/8/JEP-2019-093). The patients/participants provided their written informed consent to participate in this study. Written informed consent was obtained from the individual(s) for the publication of any potentially identifiable images or data included in this article.

## Author Contributions

LC and JTYL conceptualized the study. LC, JTYL, RP, YN, KP, QC, JS, and SB were involved in the design of the study. JTYL, LC, KP, and SS analyzed the data. JTYL drafted the manuscript which all authors reviewed and approved the final draft for submission.

## Conflict of Interest

The authors declare that the research was conducted in the absence of any commercial or financial relationships that could be construed as a potential conflict of interest.

## Publisher’s Note

All claims expressed in this article are solely those of the authors and do not necessarily represent those of their affiliated organizations, or those of the publisher, the editors and the reviewers. Any product that may be evaluated in this article, or claim that may be made by its manufacturer, is not guaranteed or endorsed by the publisher.
