# Supplementary material for: Effectiveness of a Malaysian Media Intervention Workshop: Safe Reporting on Suicide
Source: Front Psychol. 2021 Dec 15;12:666027. doi: 10.3389/fpsyg.2021.666027 (PMC8715920; doi:10.3389/fpsyg.2021.666027)
Supplement: Supplementary file 1 [file Data_Sheet_1.pdf]

Question items with significant findings	Components of Characteristics of Participants	Pre-intervention scores	Post-intervention scores	Intervention Effects	
		Median (Range)	Median (Range)	Z	*p* value
Reports about suicide should include contents of any suicide note.					
Type	None	4.50 (2-5)	5.00 (3-5)	-0.816	0.414
	Online	3.00 (2-5)	4.00 (2-5)	-2.226	0.026*
	Non-online	3.50 (3-4)	5.00 (5-5)	-1.342	0.180
	Mixed	3.50 (3-5)	4.00 (3-5)	-0.577	0.564
Role	Practitioner	3.00 (2-5)	4.00 (2-5)	-2.636	0.008*
	Non practitioner	5.00 (2-5)	5.00 (4-5)	-0.816	0.414
Job scope	Full time	4.00 (2-5)	4.00 (2-5)	-2.433	0.015*
	Part time	4.50 (2-5)	5.00 (4-5)	-1.342	0.180
Length of experience	Less than 5 years	4.00 (2-5)	5.00 (2-5)	-1.134	0.257
	5 to 10 years	3.00 (2-5)	4.00 (3-5)	-1.994	0.046*
	More than 10 years	4.00 (2-5)	4.50 (3-5)	-1.511	0.131
Frequency of encountering story on suicide	Never	4.00 (3-5)	5.00 (3-5)	-1.633	0.102
	Rarely	4.00 (2-5)	4.00 (2-5)	-0.000	1.000
	Occasionally	3.00 (2-5)	5.00 (3-5)	-2.401	0.016*
	Often	4.00 (2-5)	4.00 (3-5)	-0.447	0.655
Awareness of existing media guidelines	Yes	4.00 (2-5)	4.00 (3-5)	-2.153	0.031*
	No	3.50 (2-5)	4.50 (3-5)	-1.511	0.131
Awareness of existing Malaysian guidelines	Yes	3.00 (2-5)	4.00 (3-5)	-1.857	0.063

	No	4.00 (2-5)	5.00 (2-5)	-2.072	0.038*
Frequency of practising guidelines	Never	4.00 (2-5)	5.00 (3-5)	-1.414	0.157
	Rarely	4.50 (3-5)	4.00 (3-5)	-1.000	0.317
	Occasionally	3.00 (2-5)	5.00 (2-5)	-1.841	0.066
	Often	3.00 (3-5)	4.00 (3.5)	-1.730	0.084
Agreeableness of suicide reporting being regulated	Yes	4.00 (2-5)	5.00 (2-5)	-2.804	0.005*
	No	4.00 (3-5)	4.00 (3-5)	0.000	1.000
Media community with significant others having suicidal thoughts	Yes	4.00 (2-5)	4.00 (2-5)	-0.816	0.414
	No	3.00 (2-5)	4.00 (3-5)	-2.599	0.009*
Media community with significant others attempted suicide and survived	Yes	4.00 (2-5)	3.00 (3-4)	-0.557	0.564
	No	4.00 (2-5)	5.00 (2-5)	-2.970	0.003*
Headlines should include the method of suicide.					
Type	None	4.00 (3-5)	5.00 (4-5)	-1.890	0.059
	Online	4.00 (3-5)	4.50 (3-5)	-1.265	0.206
	Non-online	5.00 (5-5)	5.00 (5-5)	0.000	1.000
	Mixed	4.00 (2-5)	4.00 (3-5)	-1.134	0.257
Role	Practitioner	4.00 (2-5)	4.50 (3-5)	-1.706	0.088
	Non practitioner	4.00 (3-5)	5.00 (4-5)	-1.890	0.059
Job scope	Full time	4.00 (2-5)	5.00 (3-5)	-2.546	0.011*
	Part time	4.50 (4-5)	4.50 (4-5)	0.000	1.000
Length of experience	Less than 5 years	5.00 (4-5)	5.00 (4-5)	-1.000	0.317
	5 to 10 years	4.00 (3-5)	4.50 (3-5)	-0.632	0.527
	More than 10 years	4.00 (2-5)	5.00 (3-5)	-2.460	0.014*
Frequency of encountering story on suicide	Never	5.00 (2-5)	5.00 (3-5)	-0.816	0.414

	Rarely	5.00 (4-5)	4.50 (4-5)	0.000	1.000
	Occasionally	4.00 (2-5)	5.00 (3-5)	-2.486	0.013*
	Often	4.00 (4-5)	4.00 (4-5)	0.000	1.000
Awareness of existing media guidelines	Yes	4.00 (2-5)	5.00 (3-5)	-2.653	0.008*
	No	5.00 (3-5)	5.00 (4-5)	-0.816	0.414
Awareness of existing Malaysia guidelines	Yes	4.00 (2-5)	4.00 (3-5)	-1.518	0.129
	No	4.00 (3-5)	5.00 (4-5)	-1.941	0.052
Frequency of practising guidelines	Never	5.00 (4-5)	5.00 (4-5)	-0.577	0.564
	Rarely	4.00 (4-5)	4.50 (4-5)	-1.000	0.317
	Occasionally	4.00 (2-5)	4.50 (3-5)	-1.633	0.102
	Often	4.00 (2-5)	5.00 (3-5)	-1.406	0.160
Agreeableness of suicide reporting being regulated	Yes	4.00 (2-5)	5.00 (3-5)	-2.140	0.032*
	No	4.00 (3-5)	5.00 (3-5)	-1.414	0.157
Media community with significant others having suicidal thoughts	Yes	4.00 (4-5)	5.00 (3-5)	-0.447	0.655
	No	4.00 (2-5)	5.00 (3-5)	-2.495	0.013*
Media community with significant others attempted suicide and survived	Yes	5.00 (4-5)	5.00 (5-5)	-1.000	0.317
	No	4.00 (2-5)	5.00 (3-5)	-2.296	0.022*
Headlines should include the location of suicide.					
Type	None	3.50 (2-5)	4.00 (3-5)	-1.890	0.059
	Online	3.50 (2-5)	4.00 (3-5)	-2.126	0.033*
	Non-online	5.00 (5-5)	5.00 (5-5)	0.000	1.000
	Mixed	3.00 (2-4)	3.00 (3-5)	-1.342	0.180
Role	Practitioner	4.00 (2-5)	4.00 (3-5)	-2.517	0.012*
	Non practitioner	4.00 (2-5)	4.00 (3-5)	-1.633	0.102

Job scope	Full time	4.00 (2-5)	4.00 (3-5)	-2.676	0.007*
	Part time	3.50 (2-5)	4.50 (3-5)	-1.342	0.180
Length of experience	Less than 5 years	5.00 (2-5)	5.00 (4-5)	-1.342	0.180
	5 to 10 years	3.50 (2-5)	4.00 (3-5)	-1.000	0.317
	More than 10 years	3.00 (2-4)	4.00 (3-5)	-2.714	0.007*
Frequency of encountering story on suicide	Never	4.00 (2-5)	5.00 (3-5)	-1.414	0.157
	Rarely	4.00 (3-5)	4.00 (4-5)	-0.577	0.564
	Occasionally	3.00 (2-4)	4.00 (3-5)	-2.652	0.008*
	Often	4.00 (2-5)	4.00 (3-5)	-1.000	0.317
Awareness of existing media guidelines	Yes	3.00 (2-5)	4.00 (3-5)	-2.653	0.008*
	No	3.50 (2-5)	4.00 (3-5)	-2.121	0.034*
Awareness of existing Malaysia guidelines	Yes	3.00 (2-5)	3.00 (3-5)	-1.633	0.102
	No	4.00 (2-5)	4.00 (3-5)	-2.652	0.008*
Frequency of practising guidelines	Never	4.00 (2-5)	5.00 (3-5)	-1.890	0.059
	Rarely	3.50 (3-5)	4.00 (3-5)	-1.000	0.317
	Occasionally	3.50 (2-5)	4.00 (3-5)	-1.890	0.059
	Often	4.00 (2-5)	4.00 (3-5)	-1.134	0.257
Agreeableness of suicide reporting being regulated	Yes	4.00 (2-5)	4.00 (3-5)	-2.841	0.005*
	No	4.00 (3-4)	4.00 (3-5)	-1.414	0.157
Media community with significant others having suicidal thoughts	Yes	4.00 (2-5)	4.00 (3-5)	-1.265	0.206
	No	3.00 (2-5)	4.00 (3-5)	-2.972	0.003*
Media community with significant others attempted suicide and survived	Yes	5.00 (4-5)	5.00 (5-5)	-1.000	0.317
	No	3.00 (2-5)	4.00 (3-5)	-2.982	0.003*

A suspected suicide should be reported as suicide even before the cause of death is confirmed.

Type	None	5.00 (3-5)	5.00 (4-5)	1.000	0.317
	Online	4.00 (2-5)	5.00 (4-5)	-2.714	0.007*
	Non-online	5.00 (5-5)	5.00 (5-5)	0.000	1.000
	Mixed	4.00 (2-5)	4.00 (3-5)	-1.414	0.157
Role	Practitioner	4.00 (2-5)	5.00 (3-5)	-3.051	0.002*
	Non practitioner	5.00 (4-5)	5.00 (4-5)	0.000	1.000
Job scope	Full time	4.00 (2-5)	5.00 (3-5)	-2.714	0.007*
	Part time	4.50 (4-5)	5.00 (5-5)	-1.414	0.157
Length of experience	Less than 5 years	5.00 (4-5)	5.00 (4-5)	-1.000	0.317
	5 to 10 years	4.00 (4-5)	5.00 (4-5)	-2.236	0.025*
	More than 10 years	4.50 (2-5)	5.00 (3-5)	-1.890	0.059
Frequency of encountering story on suicide	Never	5.00 (2-5)	5.00 (3-5)	-1.414	0.157
	Rarely	4.00 (3-5)	5.00 (4-5)	-1.732	0.083
	Occasionally	4.50 (2-5)	5.00 (3-5)	-2.236	0.025*
	Often	4.00 (2-5)	5.00 (4-5)	-1.000	0.317
Awareness of existing media guidelines	Yes	4.00 (2-5)	5.00 (3-5)	-2.333	0.020*
	No	4.50 (3-5)	5.00 (4-5)	-1.732	0.083
Awareness of existing Malaysia guidelines	Yes	4.00 (2-5)	4.00 (3-5)	-1.890	0.059
	No	5.00 (3-5)	5.00 (4-5)	-2.646	0.008*
Frequency of practising guidelines	Never	5.00 (4-5)	5.00 (4-5)	-1.414	0.157
	Rarely	4.50 (3-5)	4.50 (4-5)	-1.000	0.317
	Occasionally	4.50 (2-5)	5.00 (3-5)	-1.633	0.102
	Often	4.00 (2-5)	5.00 (3-5)	-2.236	0.025*
Agreeableness of suicide reporting being regulated	Yes	4.00 (2-5)	5.00 (3-5)	-3.051	0.002*
	No	4.00 (3-5)	4.00 (4-5)	-1.000	0.317

Media community with significant others having suicidal thoughts	Yes	4.00 (3-5)	5.00 (4-5)	-1.732	0.083
	No	4.00 (2-5)	5.00 (3-5)	-2.714	0.007*
Media community with significant others attempted suicide and survived	Yes	5.00 (4-5)	5.00 (4-5)	0.000	1.000
	No	4.00 (2-5)	5.00 (3-5)	-3.207	0.001*
Suicide-related stories are written to cater to readers' interest.					
Type	None	2.00 (2-5)	3.00 (1-5)	-1.000	0.317
	Online	2.00 (1-5)	3.50 (2-5)	-2.588	0.010*
	Non-online	3.00 (3-3)	2.00 (2-2)	-1.414	0.157
	Mixed	2.50 (2-4)	3.00 (2-4)	-0.378	0.705
Role	Practitioner	2.00 (1-5)	3.00 (2-5)	-2.000	0.046*
	Non practitioner	2.00 (2-5)	2.00 (1-5)	-0.577	0.564
Job scope	Full time	2.00 (1-5)	3.00 (1-5)	-1.363	0.173
	Part time	2.00 (2.5)	4.00 (3-5)	-1.633	0.102
Length of experience	Less than 5 years	2.00 (1-5)	2.00 (2-5)	-0.378	0.705
	5 to 10 years	2.50 (1-5)	3.50 (2-5)	-1.725	0.084
	More than 10 years	2.00 (2-4)	3.00 (1-4)	-1.127	0.260
Frequency of encountering story on suicide	Never	4.00 (2-5)	4.00 (2-5)	-0.378	0.705
	Rarely	2.00 (1-3)	2.00 (2-4)	-0.816	0.414
	Occasionally	2.00 (2-4)	3.50 (1-5)	-1.727	0.084
	Often	2.50 (1-4)	4.00 (2-5)	-1.000	0.317
Awareness of existing media guidelines	Yes	2.50 (1-5)	3.00 (2-5)	-1.925	0.054
	No	2.00 (2-5)	3.50 (1-5)	-1.131	0.258
Awareness of existing Malaysia guidelines	Yes	3.50 (2-5)	3.00 (2-5)	-0.378	0.705

	No	2.00 (1-5)	3.00 (1-5)	-2.184	0.029*
Frequency of practising guidelines	Never	2.00 (2-5)	4.00 (2-5)	-1.890	0.059
	Rarely	2.00 (2-2)	2.00 (2-4)	-1.000	0.317
	Occasionally	3.00 (1-5)	2.50 (1-5)	0.000	1.000
	Often	3.00 (1-4)	3.00 (2-5)	-0.877	0.380
Agreeableness of suicide reporting being regulated	Yes	2.00 (1-5)	3.00 (1-5)	-1.885	0.059
	No	2.00 (2-5)	4.00 (2-5)	-1.342	0.180
Media community with significant others having suicidal thoughts	Yes	2.00 (1-4)	2.00 (2-5)	-1.552	0.121
	No	2.00 (2-5)	3.00 (1-5)	-1.554	0.120
Media community with significant others attempted suicide and survived	Yes	2.00 (1-2)	2.00 (2-5)	-1.000	0.317
	No	2.00 (1-5)	3.00 (1-5)	-1.979	0.048*
A cause for suicide should always be reported in cases of suicide.					
Type	None	3.00 (1-5)	4.50 (2-5)	-2.041	0.041*
	Online	2.00 (1-5)	3.00 (2-5)	-2.309	0.021*
	Non-online	3.00 (3-3)	3.00 (3-3)	0.000	1.000
	Mixed	2.50 (2-4)	2.50 (1-4)	-1.000	0.317
Role	Practitioner	2.50 (1-5)	3.00 (1-5)	-1.941	0.052
	Non practitioner	4.00 (1-5)	5.00 (2-5)	-1.841	0.066
Job scope	Full time	3.00 (1-5)	3.00 (1-5)	-2.389	0.017*
	Part time	2.50 (2-5)	3.00 (3-5)	-1.414	0.157
Length of experience	Less than 5 years	2.00 (1-3)	3.00 (2-5)	-2.121	0.034*
	5 to 10 years	3.00 (2-5)	3.00 (2-5)	-1.667	0.096
	More than 10 years	3.00 (1-5)	3.00 (1-5)	-0.816	0.414
Frequency of encountering story on suicide	Never	3.00 (2-5)	4.00 (2-5)	-1.604	0.109

	Rarely	2.00 (1-4)	3.00 (1-4)	-1.414	0.157
	Occasionally	3.00 (1-5)	3.00 (2-5)	-1.667	0.096
	Often	2.00 (2-2)	2.00 (2-3)	-1.000	0.317
Awareness of existing media guidelines	Yes	3.00 (1-5)	3.00 (2-5)	-2.124	0.034*
	No	2.00 (1-5)	3.00 (1-5)	-1.730	0.084
Awareness of existing Malaysia guidelines	Yes	2.00 (2-4)	3.00 (2-5)	-1.604	0.109
	No	3.00 (1-5)	3.00 (1-5)	-2.352	0.019*
Frequency of practising guidelines	Never	2.00 (2-5)	3.00 (1-5)	-1.342	0.180
	Rarely	3.00 (1-5)	4.00 (2-5)	-1.342	0.180
	Occasionally	2.00 (1-4)	3.00 (2-5)	-1.841	0.066
	Often	3.00 (2-3)	3.00 (2-3)	-0.577	0.564
Agreeableness of suicide reporting being regulated	Yes	2.00 (1-5)	3.00 (2-5)	-2.949	0.003*
	No	3.00 (2-4)	3.00 (1-4)	-0.447	0.655
Media community with significant others having suicidal thoughts	Yes	2.00 (1-4)	3.00 (2-5)	-2.449	0.014*
	No	3.00 (1-5)	3.00 (1-5)	-1.997	0.046*
Media community with significant others attempted suicide and survived	Yes	2.00 (1-2)	3.00 (2-3)	-1.732	0.083
	No	3.00 (1-5)	3.00 (1-5)	-2.437	0.015*
Reports should include the location of a suicide death.					
Type	None	3.00 (2-5)	4.00 (3-5)	-1.890	0.059
	Online	3.00 (2-5)	4.00 (2-5)	-2.489	0.013*
	Non-online	3.00 (2-4)	5.00 (5-5)	-1.342	0.180
	Mixed	2.50 (2-3)	2.50 (2-3)	0.000	1.000
Role	Practitioner	3.00 (2-5)	3.50 (2-5)	-2.719	0.007*
	Non practitioner	3.00 (2-5)	4.00 (3-5)	-1.732	0.083

Job scope	Full time	3.00 (2-5)	3.00 (2-5)	-3.092	0.002*
	Part time	3.00 (2-5)	4.00 (2-5)	-0.816	0.414
Length of experience	Less than 5 years	4.00 (2-5)	5.00 (3-5)	-1.841	0.066
	5 to 10 years	3.00 (2-5)	3.50 (2-5)	-1.890	0.059
	More than 10 years	3.00 (2-4)	3.00 (2-5)	-1.725	0.084
Frequency of encountering story on suicide	Never	3.00 (2-5)	4.00 (2-5)	-0.816	0.414
	Rarely	3.00 (2-4)	3.50 (2-5)	-1.134	0.257
	Occasionally	2.50 (2-4)	3.50 (2-5)	-2.810	0.005*
	Often	3.00 (2-4)	5.00 (3-5)	-1.342	0.180
Awareness of existing media guidelines	Yes	3.00 (2-5)	4.00 (2-5)	-2.913	0.004*
	No	2.50 (2-5)	4.00 (2-5)	-1.406	0.160
Awareness of existing Malaysia guidelines	Yes	3.00 (2-5)	3.00 (2-5)	-1.633	0.102
	No	3.00 (2-5)	4.00 (2-5)	-2.858	0.004*
Frequency of practising guidelines	Never	4.00 (2-5)	4.00 (2-5)	-1.000	0.317
	Rarely	3.00 (2-4)	3.50 (3-4)	-1.000	0.317
	Occasionally	3.00 (2-5)	4.00 (2-5)	-1.890	0.059
	Often	2.00 (2-4)	3.00 (2-5)	-2.264	0.024*
Agreeableness of suicide reporting being regulated	Yes	3.00 (2-5)	4.00 (2-5)	-3.345	0.001*
	No	3.00 (2-3)	3.00 (2-4)	-0.447	0.655
Media community with significant others having suicidal thoughts	Yes	3.00 (2-4)	4.00 (3-5)	-2.460	0.014*
	No	3.00 (2-5)	3.00 (2-5)	-2.230	0.026*
Media community with significant others attempted suicide and survived	Yes	4.00 (4-4)	5.00 (4-5)	-1.414	0.157
	No	3.00 (2-5)	3.00 (2-5)	-3.011	0.003*

People in the media can be negatively affected when reporting on suicide.

Type	None	4.00 (2-5)	4.00 (2-5)	-0.447	0.655
	Online	4.00 (3-5)	5.00 (4-5)	-2.449	0.014*
	Non-online	4.00 (4-4)	4.50 (4-5)	-1.000	0.317
	Mixed	3.50 (2-5)	4.00 (2-4)	0.000	1.000
Role	Practitioner	4.00 (2-5)	4.00 (2-5)	-2.111	0.035*
	Non practitioner	4.00 (2-5)	4.00 (2-5)	-0.447	0.655
Job scope	Full time	4.00 (2-5)	4.00 (2-5)	-2.000	0.046*
	Part time	4.50 (4-5)	4.50 (4-5)	0.000	1.000
Length of experience	Less than 5 years	4.00 (3-5)	4.00 (2-5)	-1.000	0.317
	5 to 10 years	4.00 (3-5)	4.50 (3-5)	-1.342	0.180
	More than 10 years	4.00 (2-5)	4.00 (2-5)	-1.134	0.257
Frequency of encountering story on suicide	Never	4.00 (2-5)	4.00 (2-5)	-1.414	0.157
	Rarely	4.00 (3-5)	4.00 (2-5)	0.000	1.000
	Occasionally	4.00 (2-5)	5.00 (3-5)	-1.667	0.096
	Often	4.00 (3-5)	4.00 (4-5)	-1.000	0.317
Awareness of existing media guidelines	Yes	4.00 (2-5)	4.00 (2-5)	-1.667	0.096
	No	4.00 (2-5)	4.00 (4-5)	-0.816	0.414
Awareness of existing Malaysia guidelines	Yes	4.00 (2-4)	4.00 (2-5)	-1.342	0.180
	No	4.00 (2-5)	5.00 (2-5)	-1.508	0.132*
Frequency of practising guidelines	Never	5.00 (3-5)	4.00 (4-5)	0.000	1.000
	Rarely	4.00 (3-5)	4.00 (2-5)	-1.000	0.317
	Occasionally	4.00 (2-4)	4.00 (4-5)	-1.890	0.059
	Often	4.00 (2-5)	5.00 (2-5)	-1.342	0.180
Agreeableness of suicide reporting being regulated	Yes	4.00 (2-5)	4.00 (2-5)	-1.941	0.052
	No	4.00 (3-5)	4.00 (4-5)	-0.577	0.564

Media community with significant others having suicidal thoughts	Yes	4.00 (3-5)	4.00 (2-5)	-0.447	0.655
	No	4.00 (2-5)	4.00 (2-5)	-2.111	0.035*
Media community with significant others attempted suicide and survived	Yes	3.00 (3-5)	4.00 (2-5)	0.000	1.000
	No	4.00 (2-5)	4.00 (2-5)	-2.138	0.033*

**p*<.05 significant findings

Supplementary Material: Characteristics of participants contributing to the significant findings in each question item.
